# Raster image cross-correlation analysis for spatiotemporal visualization of intracellular degradation activities against exogenous DNAs

**DOI:** 10.1038/srep14428

**Published:** 2015-09-24

**Authors:** Akira Sasaki, Johtaro Yamamoto, Takashi Jin, Masataka Kinjo

**Affiliations:** 1 Biomedical Research Institute, National Institute of Advanced Industrial Science and Technology (AIST), Tsukuba, Ibaraki 305-8566, Japan; 2 Laboratory for Nano-Bio Probes, RIKEN Quantitative Biology Center (QBiC), Suita, Osaka 565-0874, Japan; 3 Laboratory of Molecular Cell Dynamics, Faculty of Advanced Life Science, Hokkaido University, Sapporo, Hokkaido 001-0021, Japan; 4 WPI Immunology Frontier Research Center, Osaka University, Suita, Osaka 565-0871, Japan; 5 Graduate School of Frontier Biosciences, Osaka University, Suita, Osaka 565-0871, Japan

## Abstract

Reducing intracellular DNA degradation is critical to enhance the efficiency of gene therapy. Exogenous DNA incorporation into cells is strictly blocked by the defense machinery of intracellular nuclease activity. Raster image correlation spectroscopy (RICS) and raster image cross-correlation spectroscopy (cross-correlation RICS; ccRICS) are image-based correlation methods. These powerful tools allow the study of spatiotemporal molecular dynamics. Here we performed spatiotemporal ccRICS analyses of fluorescent DNA and directly monitored the process of exogenous DNA degradation in living cell cytoplasm. Such direct monitors of DNA degradation allow us to determine the fate of the exogenous DNA in living cells. On comparing the process in living cells, our study shows that cytoplasmic nuclease activity differs between cell lines; therefore, we propose that the difference of nuclease activity in cytoplasm dictates a different resistance to exogenous DNA incorporation. New insight on efficient gene delivery can be provided with our study.

Gene therapy^1^, which promotes expression of target proteins or knockdown of genes, is considered for use in refractory diseases such as Parkinson’s disease^1^,^2^,^3^, Alzheimer’s disease^1^,^4^, and also cancer^1^. An important issue limiting the clinical application of gene therapy is the poor expression efficiency of exogenous genes, particularly regarding the use of nonviral gene carriers^5^,^6^. To improve the efficiency, the incorporated exogenous DNAs should be effectively transferred into the cell nucleus. By developing novel nonviral gene carriers to enhance gene expression, several researchers significantly contributed to the research field of gene delivery. However, sufficient expression efficiency has not yet been achieved. The knowledge of delivery mechanisms and pathways is limited because of the lack of a suitable technique to observe intracellular DNA behavior. In nature, the expression of exogenous genes is strictly blocked as an invasion by the defense machinery of the cell; in other words, nuclease activity inhibits artificial gene incorporation^7^ and expression of exogenous DNA. Exogenous gene expression in the context of this defense machinery requires a spatiotemporal analysis of the DNA fate incorporated into living cells.

In general, for transfection, circular plasmid vectors are used because the expression rate is drastically decreased with linear DNAs^8^. DNA transfection efficiency is also different between cell lines^9^. For example, the expression efficiency of MEF cells is relatively low^10^; in contrast, HEK293 cells can be transfected efficiently and the cell line is usually used for protein factory in mammalian cells^11^. Previously, we have monitored nuclease activity in living cells using fluorescence correlation spectroscopy (FCS)^12^,^13^,^14^ and fluorescence cross-correlation spectroscopy (FCCS)^14^,^15^. The results suggested that exonuclease activity plays an important role in cytoplasmic DNA degradation, affecting the expression efficiency of incorporated DNAs^14^. The limitation of FCS/FCCS techniques is that only one point measurements are possible and different regions of interest in cells cannot be simultaneously compared. Because DNA degradation progresses on the minute time scale^13^,^14^, we aimed to establish a quantitative method to visualize transportation and nuclease degradation of exogenous DNAs in living cells.

Raster image correlation spectroscopy (RICS)^16^,^17^,^18^,^19^ and raster image cross-correlation spectroscopy (cross-correlation RICS; ccRICS)^20^ are image-based correlation spectroscopy techniques and are powerful tools for studying spatiotemporal molecular dynamics. RICS enables to extract molecular dynamics information from fluctuations of fluorescence intensity recorded in raster-scanned fluorescence images. ccRICS is the dual-color extension of RICS, which detects the interaction between two fluorescent-labeled molecules by the coincident fluctuation signals at different detection channels. The advantages of RICS/ccRICS are that dynamic molecular information can be extracted from the image of confocal laser scanning microscopy (LSM), cells can be continuously imaged throughout the measurement, and regions of interest can be selected after image acquisition. This spatiotemporal analysis is ideal for investigating molecular dynamics, reaction kinetics, and molecular interactions in living cells.

In this work, the dual-color ccRICS technique was employed to visualize when and where the exogenous DNA injected into living cell cytoplasm is degraded. Such direct measurements of DNA degradation allow us to determine the fate of the exogenous DNA in a timely manner in living cells and to monitor the cytoplasmic nuclease activity, which is the critical factor for efficient gene delivery.

## Results

### Expression efficiency reduction with linearized DNA

**Figure 1:**
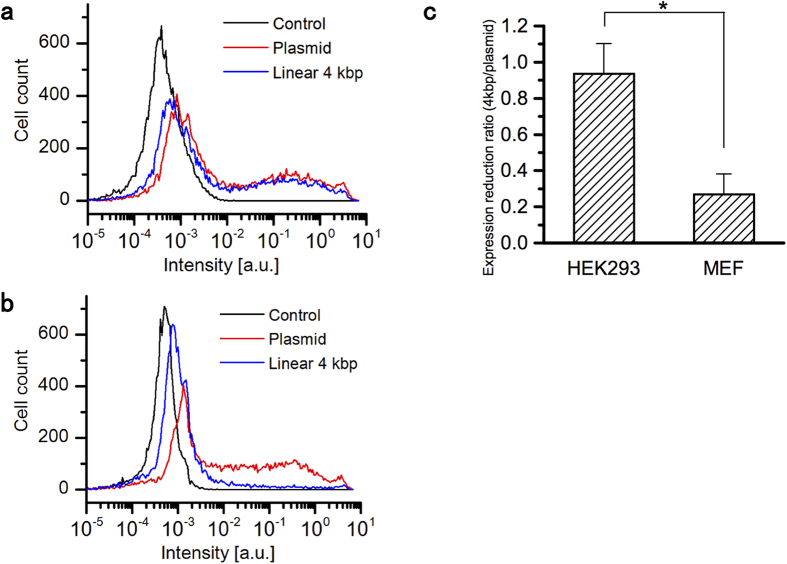
EGFP expression analysis by flow cytometer. Histograms of EGFP expression rate in (**a**) HEK293 and (**b**) MEF cells transfected with the pEGFP-C1 plasmid or linear 4-kbp DNA. Cells without transfection were tested as a background fluorescence intensity control. (**c**) Expression reduction ratio in the case of plasmid and linear 4-kbp DNA transfection. (P = 0.0068, Student’s t-test).

In our previous study, we found that the effect of DNA linearization for expression efficiency was different between cell lines by bulk biochemical analysis^14^. To confirm the effect in MEF and HEK293 cell lines, an enhanced green fluorescent protein (EGFP) expression assay in single cells was conducted with a flow cytometer. We synthesized a 4-kbp linear DNA, containing an EGFP coding region, by PCR. The same amount of pEGFP-C1 plasmid and linear DNA generated by PCR were transfected into MEF and HEK293 cells. Expression rates of EGFP in single cells were analyzed by flow cytometry. There was a small difference of EGFP expression distribution between the circular plasmid and linear DNA in HEK293 cells (Fig. 1a). On the other hand, high EGFP expression was decreased with the linear DNA compared with the circular plasmid in MEF cells (Fig. 1b). Cell samples that did not undergo DNA transfection were tested to estimate the background autofluorescence (control in Fig. 1a,b). Figure 1c shows the fold difference of cells whose fluorescence intensity is higher than the autofluorescence signal, 10^−2.5^ (a. u.), in the experiments presented in Fig. 1a,b (Expression reduction ratio). Expression efficiency in MEF cells is significantly decreased by 3-fold in the linear 4-kbp DNA compared with that in the plasmid. The P value was 0.0068 (n = 3) when MEF and HEK293 cells are compared.

### Cross-correlation analysis of dual color-fluorescent DNA probe in solution

**Figure 2:**
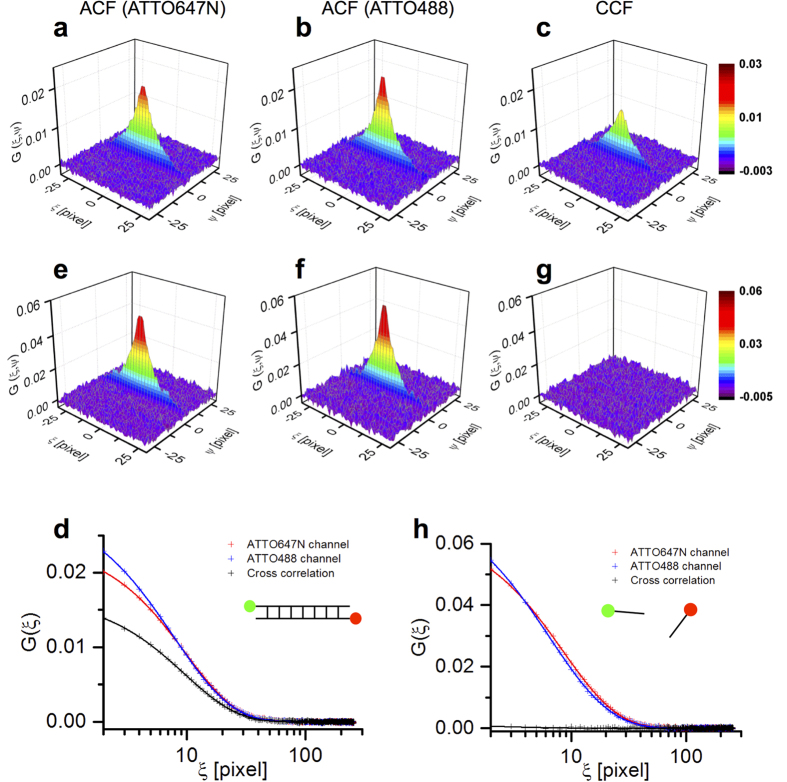
Correlation functions of DNA probes obtained by ccRICS in solution. Autocorrelation functions (ACF) of (**a**) the red channel, (**b**) green channel, and (**c**) cross-correlation function (CCF) of linear 30-bp DNA probes labeled with ATTO488 and ATTO647N at each 5′ ends. (**d**) Merged graph of *G*(*ξ* > 0, *ψ* = 0) of auto- and cross-correlation functions of 30-bp DNA. Cross symbols show the raw correlation data and lines show fitting result. (**e**–**g**,**h**) Data set for the fluorescent-labeled oligonucleotide mix used as a negative control.

Linear 30-bp DNA labeled with ATTO488 and ATTO647N on both 5′-ends was prepared as a 100 nM solution and was imaged on the coverglass by confocal microscopy for 385 s (50 images). The freely diffused fluorescent DNA molecules were detected in a focal plane positioned 200 μm above the glass surface. As a negative control, a 5′ fluorescence-labeled (ATTO488 or ATTO647N) single-strand oligonucleotide mixture was measured under the same conditions. Two-dimensional spatial autocorrelation functions (ACF, Fig. 2a,b,e,f) and cross-correlation functions (CCF, Fig. 2c,g) were obtained by ccRICS analysis. The *G*(*ξ*, 0) region of ACFs and CCF were extracted and fitted by model equation (Fig. 2d,h). To assess the degree of cross-correlation amplitude, relative cross-correlation amplitude (RCA) was calculated. RCA was 0.686 for the 30-bp double-labeled DNA and 0.024 for the oligonucleotide mix negative control. The values represent the upper and lower limits of the fraction of DNAs that have ATTO488 and ATTO647N fluorophores at both DNA ends, respectively; thus, they are intact DNAs and completely digested DNA.

**Figure 3:**
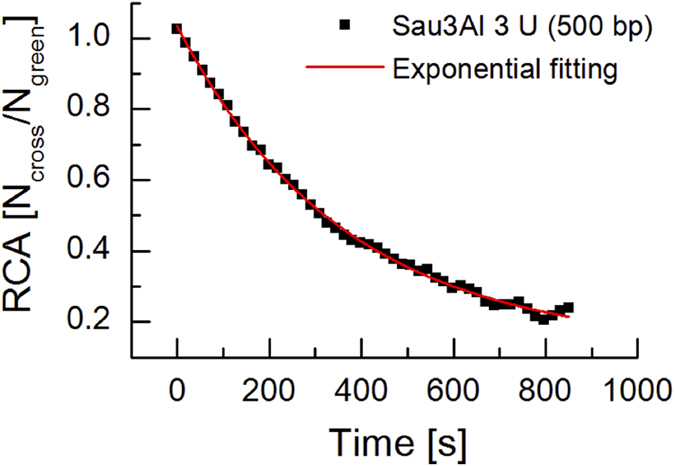
Degradation kinetics of the 500-bp DNA probe by the Sau3A I restriction enzyme monitored by ccRICS in solution. Black squares indicate RCA at each time point and red line indicated the exponential fitting curve, respectively.

To confirm whether ccRICS can evaluate the degradation kinetics of DNA probes, we continuously monitored the degradation of fluorescent DNA (ATTO488-500-bp-ATTO647N) with the restriction endonuclease Sau3A I in solution. The 500-bp DNA was designed to have a single restriction enzyme recognition site. The RCA value was decreased in an exponential manner by adding 3 units of Sau3A I enzyme (Fig. 3). This result confirmed that we can monitor the time-dependent degradation of DNA using ccRICS analysis.

### Spatiotemporal degradation analysis in living cells

**Figure 4:**
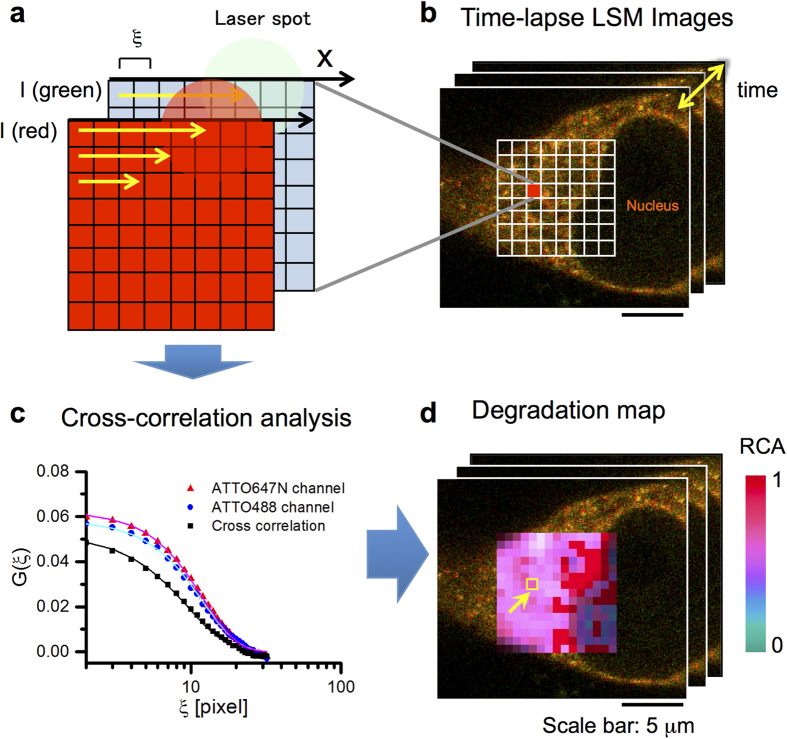
Schematic diagram of ccRICS data acquisition and analysis procedure. ROIs (**a**) in the time-lapse LSM images of a living cell (**b**) are defined, and a cross-correlation analysis is performed (**c**) for each ROI. Obtained RCA values are mapped on LSM images and the map is modulated by the fluorescence intensity of each pixel (**d**).

We monitored the intracellular degradation of DNA probes in MEF and HEK293 cells using time-lapse movies. Linear 30-bp DNA probes were microinjected into the cytoplasm of cells that contained a nuclear envelope (interphase). This was followed by time-lapse, dual-color LSM imaging for ccRICS analysis during the next10–60 min (Fig. 4a,b). Fluorescence fluctuations originating from molecular diffusion were extracted from the obtained LSM images (1024 × 1024 pixels) by detrending and the average fluorescence intensity was corrected to the original value. The detrended images were analyzed by a homemade ccRICS software to obtain the autocorrelation and cross-correlation functions. To generate spatiotemporal maps of the degradation activity in the cell, the images were divided into 32 × 32 ROIs (64 × 64 pixels, half-overlapping each other), and correlation analysis was conducted for each ROI (Fig. 4c). The obtained correlation functions were averaged for 10 frames, fitted by model equation, and RCA was calculated in each ROI and frame (Supplement Figs. S1–S4). The map of the RCA value was constructed and modulated by fluorescence intensity at each pixel (see Methods section). The RCA value shows the degree of DNA probe degradation in ROI so that the spatiotemporal DNA degradation activity is described by a two-dimensional map and a time-lapse movie (Fig. 4d).

**Figure 5:**
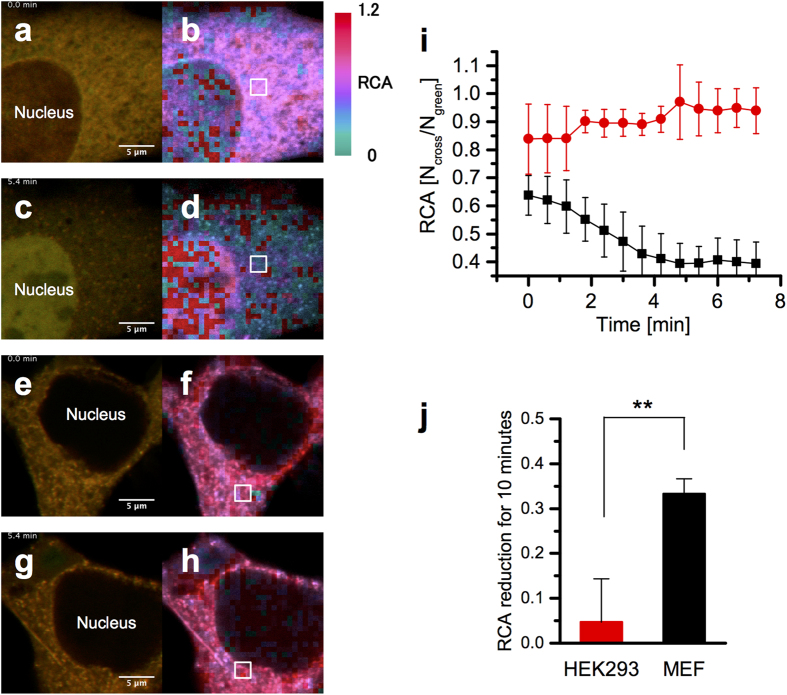
Spatiotemporal ccRICS analysis of DNA degradation in living cells. (**a**) LSM image of an MEF cell immediately right after DNA injection and (**b**) RCA degradation map of ccRICS analysis. (**c**) LSM image and (**d**) degradation map of the MEF cell 5.4 min after injection. (**e**) LSM image of a HEK293 cell immediately after DNA injection and (**f**) RCA degradation map. (**g**) LSM image and (**h**) degradation map of the HEK293 cell 5.4 min after injection. (**i**) Time dependent RCA changes in MEF (black) and in HEK293 (red) cells at the region indicated by a white square in b, d, f, and h images. Error bar shows standard deviation of nine different regions in a white square. (**j**) RCA reduction in HEK293 and MEF cells for initial 10 mins after DNA injection (P = 0.0016, Student’s t-test).

Figure 5 shows the result of the spatiotemporal ccRICS analysis of MEF and HEK293 cells. In the case of MEF cells, RCA in the cytoplasm was drastically decreased 5 min after the injection of DNA probes (pseudocolor changed from red to blue, Fig. 5a–d and Supplement Movie S1). On the other hand, the RCA value in HEK293 cells was not decreased in this timescale (Fig. 5e–h and Supplement Movie S2). The changes in average RCA value in the cell cytoplasmic regions of interest, represented as a white square with nine measurement regions, were plotted with time (Fig. 5i). The degradation of DNA was confirmed by conventional FCCS measurements 1 h after DNA injection (Supplement Fig. S5). From fluorescence images, the accumulation of fluorescence signal into the nucleus was observed in MEF cells (Fig. 5a,c). In contrast, fluorescence signal in HEK293 cells was still detected in cytoplasm (Fig. 5e,g). Figure 5j shows RCA reductions in the cytoplasmic regions of HEK293 and MEF cells during the initial 10 mins after DNA injection. The RCA in MEF cells decreased significantly during this period compared with that in HEK293 cells. The P value was 0.0016 (n = 5) when HEK293 and MEF cells are compared.

## Discussion

This study aimed to evaluate when and where exogenous DNAs are degraded in living cells. We monitored the fate of fluorescent-labeled DNA probes that were directly injected into the cytoplasm. ccRICS analysis revealed that the injected DNAs were degraded within 10 min in the cytoplasm of MEF cells and were degraded to a lesser extent in HEK293 cells. The direct measurements of DNA degradation in living cells (Fig. 5) show that the nuclease activities are drastically different in the cytoplasm of two cell lines, and the degradation activities have a considerable effect on exogenous gene delivery and expression efficiency in cells (Fig. 1). From time-lapse fluorescence imaging, the transportation of exogenous DNAs and/or DNA fragments into the cell nucleus was observed only in MEF cells (Fig. 5a,c,e,g). These results indicate that the degraded DNA fragments, generated by cytoplasmic nucleases, have become more accessible to the nucleus through the nuclear pores.

To investigate the continuously changing molecular dynamics, both time and spatial resolution are important. The ccRICS technique is based on fluorescence time-lapse imaging, so that time resolution depends on the frame rate of image acquisition. In our case, the movie of the degradation map (Supplement Movie S1, S2) was constructed at 0.6 min per frame. This time resolution was sufficient to monitor the degradation kinetics of injected DNAs (Fig. 5i). The observed timescale of exogenous DNA degradation (several minutes to hours) is shorter than that observed for conventional DNA transfection and gene expression (hours to days); this shows that avoiding degradation is a critical factor to achieve efficient gene delivery. In the case of nonviral gene delivery, the shape of the DNA/carrier complex^8^ and association and dissociation between DNAs and gene carriers^21^ will have an effect on the stability of DNAs against cytoplasmic degradation. To calculate correlation functions, the acquired images should be divided into small ROIs, and the spatial resolution of the correlation map is defined by the size of ROI. Compared with FCS/FCCS, such image-based correlation analyses have the advantage to allow monitoring of molecular dynamics and/or molecular interactions at multiple ROIs. The degradation of the DNA probes in the ROIs were simultaneously detected in a wide area of the cytoplasm. The result indicates that the DNA probes freely diffused in the cytoplasm were degraded by a cytoplasmic nuclease, not inside a vesicle such as the lysosome. We focused on the cross-correlation amplitude (RCA value), to visualize the degree of DNA degradation; however, the technique can also estimate the diffusion coefficient of DNA and degraded fragments. In our current setup, information concerning molecular number and RCA can be determined from the amplitude of the correlation curves. However, it was difficult to obtain the precise diffusional properties of DNA. Therefore, an optimal scan speed and further analytical methods, such as global analysis^22^, are needed. We considered a photophysical process, such as a triplet transition, although the phenomenon was not observed in our results because the time resolution of the correlation time under our measurement conditions was too long (12.8 μs) to detect a photophysical process (on the order of 1 μs).

In addition, we detected fluorescent foci structures in the fluorescence intensity image (Fig. 5c). The foci could correspond to a complex of DNA probes with cellular DNA sensor proteins^23^,^24^ and the complex could promote immune responses against infection.

In summary, we have directly monitored exogenous DNA degradation in living cells by ccRICS, and we propose that the difference of nuclease activity in cytoplasm impacts a cell’s resistance to exogenous DNA incorporation. This spatiotemporal analysis is useful to investigate molecular dynamics, reaction kinetics, and molecular interactions. To the best of our knowledge, this is first report by direct measurements in living cells, which shows that cytoplasmic nuclease activity differs between cell lines. This study provides new insights on efficient gene delivery by revealing the importance of cytoplasmic degradation of DNAs. Furthermore, the results indicate that different strategies for gene delivery should be applied depending on the different type of target cells or tissue. It should be noted that the nuclease involved in exogenous DNA degradation has not yet been identified, although several candidates have indicated^14^,^25^. We believe that the combination of fluorescence correlation methods and next-generation sequencing and/or mass spectrometry could solve this question.

## Methods

### Cell culture and microinjection

Mouse embryonic fibroblast^26^ (MEF, gift from Dr. Akira Kitamura, Hokkaido University) and HEK293 cells (Riken BRC, Ibaraki, Japan) were maintained in a 5% CO_2_ humidified atmosphere at 37 °C in Dulbecco’s modified Eagle’s medium (DMEM, Sigma-Aldrich, St. Louis, MO, USA) supplemented with 10% fetal bovine serum (Hyclone Lab., Logan, UT, USA), 1 × 10^5^ U l^−1^ penicillin G and 100 mg l^−1^ streptomycin sulfate (Wako, Osaka, Japan). The day before the experiment, cells (early passage) were plated on a 35-mm glass-base dish (AGC Techno Glass, Ltd., Shizuoka, Japan) to 60–70% confluence for live-cell imaging or a 6-well plate (Thermo Fisher Scientific, Waltham, MA, USA) for expression assay. The medium was replaced by phenol red-free medium (Opti-MEM, Life Technologies, Gaithersburg, MD, USA) before confocal imaging. Microinjection of the DNA probe was conducted at the confocal microscope stage by combining Femtojet (Eppendorf, Hamburg, Germany) and Injectman NI2 (Eppendorf) with Femtotip (Eppendorf) as an injection needle. Injection pressure was adjusted to deliver the desired amount of DNA.

### Expression assay

Linear DNA (4-kbp) with the EGFP coding region was synthesized by PCR using the pEGFP-C1 plasmid (Clontech, Palo Alto, CA, USA) as a template. The following sequences of primer sets (Sigma-Aldrich Japan, Hokkaido, Japan) used were:

Forward primer: 5′-CCG CTA TCA GGA CAT AGC GT-3′

Reverse primer: 5′-TTG TCT GTT GTG CCC AGT CA-3′

Cells in 6-well plates were transfected using Lipofectamine2000 (Life Technologies) with 1 μg of pEGFP-C1 plasmid or linear 4-kbp DNA per well according to the manufacturer’s instructions. The transfected cells were trypsinized and collected 24 h after transfection. The EGFP gene expressions of each cell (at least 10,000 cells) were quantified by MACSquant Analyzer (Miltenyi Biotec GmbH, Bergisch Gladbach, Germany).

### Dual color-fluorescent DNA probe

ATTO488- and ATTO647N-labeled 30-bp double-stranded DNA (DNA probe) was custom ordered at Sigma-Aldrich Japan. DNA was prepared by annealing ATTO488-5′-TAC CTT GGG GCC GGT GAG AAT TCG GCC TTT-3′ and ATTO647N-5′-AAA GGC CGA ATT CTC ACC GGC CCC AAG GTA-3′, followed by HPLC purification. Linear 500-bp DNA labeled by ATTO488 and ATTO647N was synthesized by PCR. PCR was performed with TaKaRa Ex Taq (Takara Bio Inc., Shiga, Japan; 5 U μl^−1^), dNTP Mixture (0.2 mM each), and Lambda-DNA (0.002 μg ml^−1^) as a template using the 5′-ATTO647N-labeled forward primer and the 5′-ATTO488-labeled reverse primer (0.4 μM each; Sigma-Aldrich Japan). The sequences of primers used were:

Forward primer: ATTO647N-5′-GAT GAG TTC GTG TCC GTA CAA CT-3′

Reverse primer (for 500-bp): ATTO488-5′-GGT TAT CGA AAT CAG CCA CAG CG-3′

PCR products were purified three times using MicroSpin S-400 HR columns (GE Healthcare, Uppsala, Sweden).

### Confocal imaging for raster image cross-correlation spectroscopy

Time-lapse LSM images for ccRICS analysis were acquired by a combination system consisting of an LSM510 and a ConfoCor3 (Zeiss, Jena, Germany) with a temperature control system. ATTO488 was excited by a 488-nm Ar-ion laser and ATTO647N was excited by a 633-nm He–Ne laser through a water-immersion objective (C-Apochromat, 40×, 1.2 NA; Zeiss). Two lasers simultaneously illuminated the observation volume at the same position. Emission signals were collected by photon counting at 505–610 nm for ATTO488 (green channel) and >650 nm for ATTO647N (red channel) with avalanche photodiodes (APDs). The pixel size was 22 nm, which is 5–10 times smaller than the focused laser spot, and the pixel dwell time was 5–50 μs per pixel.

### Enzyme degradation tests in solution

The DNA solution (ATTO647N-500-bp-ATTO488) was incubated with the Sau3A I restriction enzyme (3 units, Takara Bio Inc.) in a total volume of 50 μl. The incubation was conducted at 37 °C at the microscope stage. Time-lapse images of the solution during enzymatic reaction were recorded by LSM.

### ccRICS analysis

Correlation analysis^20^ of the images was performed using homemade programs written in MATLAB (MathWorks, Natick, MA, USA). In the case of heterogeneous samples, such as living cells, we applied detrending; the temporal average (three images) of the image intensity was substituted for the raw image, so that the intensity changes originating from the immobile fraction and the cell structure were removed. The obtained autocorrelation functions of the green channel *G*_*g*_(*ξ*, *ψ*), red channel *G*_*r*_(*ξ*, *ψ*), and cross-correlation function *G*_*c*_(*ξ*, *ψ*) were fitted with the following equation:


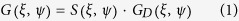


The correlation function *G*(*ξ*, *ψ*) is a function of the laser scanning term *S*(*ξ*, *ψ*) and the diffusion term *G*_*D*_(*ξ*, *ψ*) as follows:


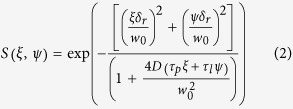



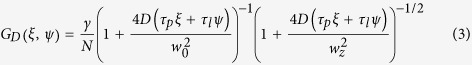


Here *δ*_*r*_ represents the pixel size, and *w*_0_ and *w*_*z*_ are the radii of lateral direction and axial direction of the focal volume, respectively. *τ*_*p*_ and *τ*_*l*_ are the pixel dwell time and the line time, respectively. *N* is the average number of molecules in the observation volume and *γ*  = 0.35 is a correction factor for a three-dimensional Gaussian illumination profile of laser illumination, and the diffusion coefficient (*D*) is obtained. The term *G*_*D*_(*ξ*, *ψ*) is assuming a three-dimensional free diffusion. Nonlinear least squares fitting was conducted only for the *ξ*-direction using MATLAB or OriginPro (OriginLab Corp., Northampton, MA, USA). From the dual-color autocorrelation analysis, the average numbers of green (*N*_*g*_) and red fluorescent particles (*N*_*r*_) are obtained. Particles with both green and red fluorescence (*N*_*gr*_) are calculated from the apparent *N*_*c*_ of the cross-correlation function using following equation:


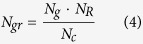


For the quantitative evaluation of cross-correlations among various samples, *N*_*gr*_ is normalized by *N*_*g*_ (relative cross-correlation amplitude; RCA). The RCA value shows the fraction of intact DNAs with both green and red fluorescence.

## Additional Information

**How to cite this article**: Sasaki, A. *et al.* Raster image cross-correlation analysis for spatiotemporal visualization of intracellular degradation activities against exogenous DNAs. *Sci. Rep.* **5**, 14428; doi: 10.1038/srep14428 (2015).

## Supplementary Material

Supplement Movie S1

Supplement Movie S2

Supplementary Information
