# Supplementary material for: Raster image cross-correlation analysis for spatiotemporal visualization of intracellular degradation activities against exogenous DNAs
Source: Sci Rep. 2015 Sep 24;5:14428. doi: 10.1038/srep14428 (PMC4585853; doi:10.1038/srep14428)
Supplement: Supplementary Information [file srep14428-s3.pdf]

Supplementary Information

Raster image cross-correlation analysis for spatiotemporal visualization of intracellular degradation activities against exogenous DNAs

Akira Sasaki[1, 2, *], Johtaro Yamamoto[3], Takashi Jin[2, 4, 5] and Masataka Kinjo[3, *]

[1]Bioanalytical Research Group, Biomedical Research Institute, National Institute of Advanced Industrial Science and Technology (AIST), Tsukuba, Ibaraki 305-8566, Japan

[2]Laboratory for Nano-Bio Probes, RIKEN Quantitative Biology Center (QBiC), Suita, Osaka 565-0874, Japan

[3]Laboratory of Molecular Cell Dynamics, Faculty of Advanced Life Science, Hokkaido University, Sapporo, Hokkaido 001-0021, Japan.

[4]WPI Immunology Frontier Research Center, Osaka University, Suita, Osaka 565-0871, Japan

[5]Graduate School of Frontier Biosciences, Osaka University, Suita, Osaka 565-0871, Japan

Supplement Movie S1.

Dual-color confocal time-lapse imaging and its correlation mapping of MEF cell. Fluorescence intensity images of green and red channels are merged (left). Degradation map of injected DNA probes (right). Frame rate is 7 frames/s.

Supplement Movie S2.

Dual-color confocal time-lapse imaging and its correlation mapping of HEK293 cell. Fluorescence intensity images of green and red channels are merged (left). Degradation map of injected DNA probes (right). Frame rate is 7 frames/s.

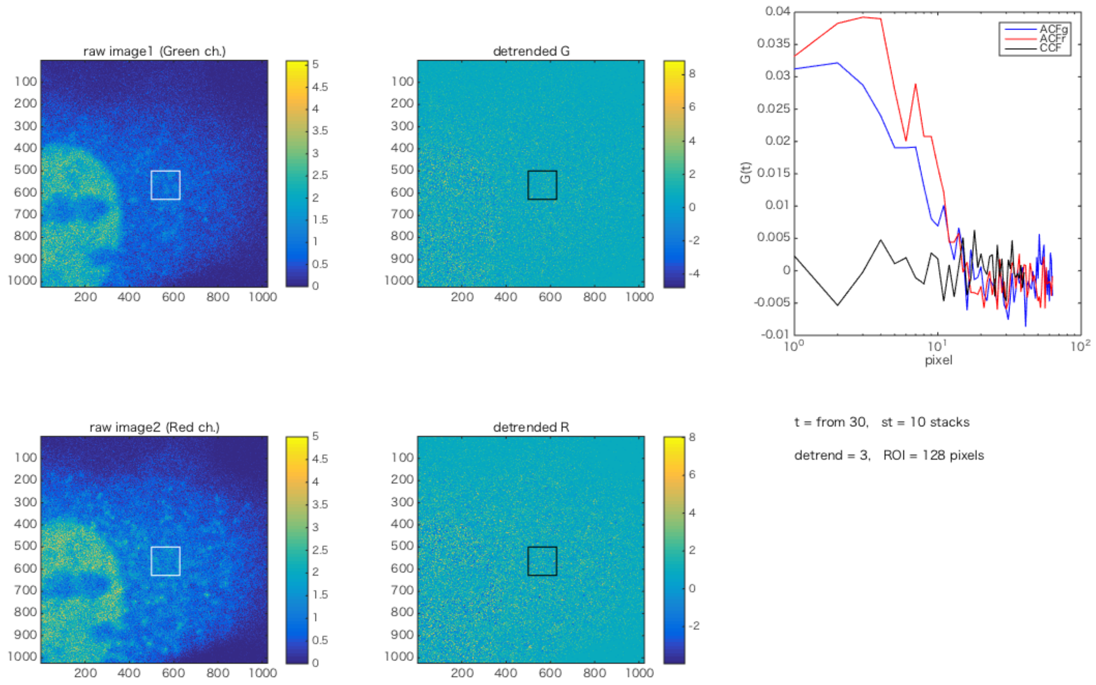

Supplement Figure S1.

LSM fluorescence intensity images of the MEF cell (green and red channel with or without detrending) 5.4 min after injection. Pseudocolor indicates photon counts at each pixel. Correlation functions of green channel, red channel, and between each channel (cross correlation) were obtained by calculating correlation in the ROI indicated as squares in the images.

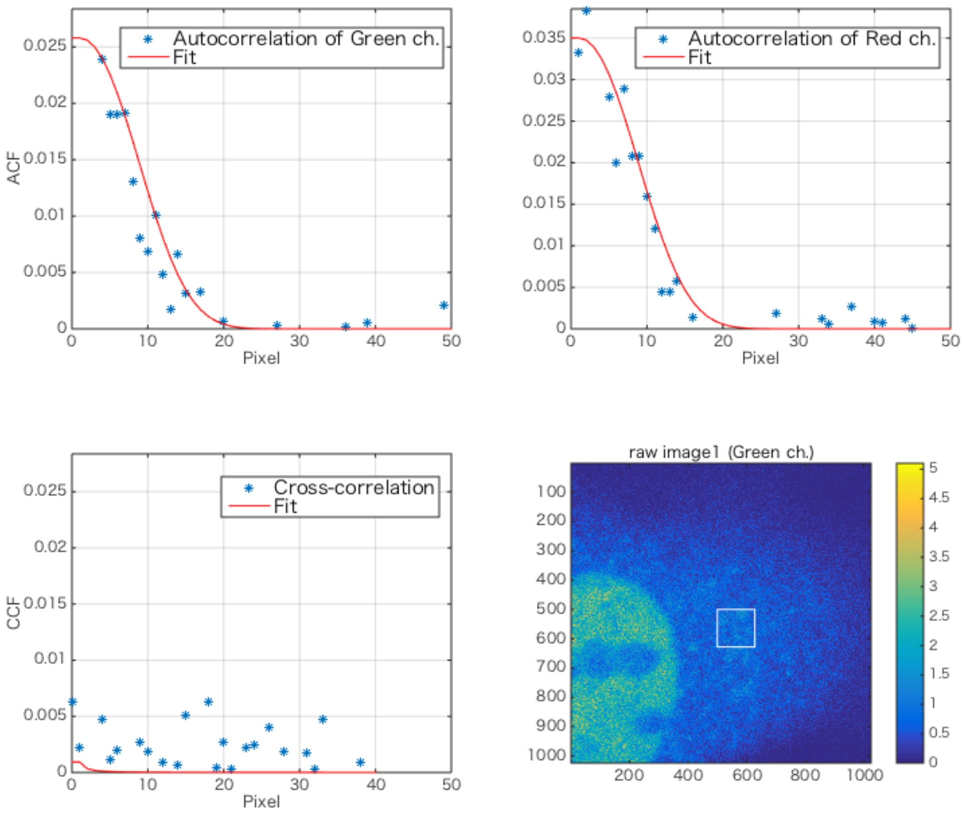

Supplement Figure S2.

Correlation functions (symbol) and its fitting results (red line) of MEF cell (5.4 min after DNA injection) in the ROI at the position of squares in the image.

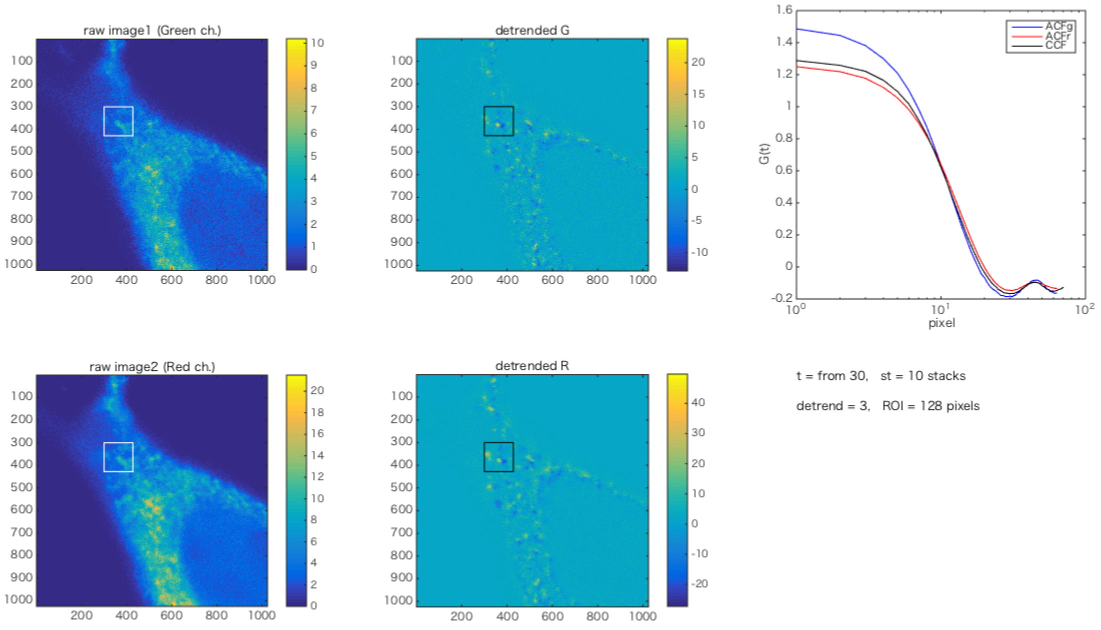

Supplement Figure S3.

LSM fluorescence intensity images of the HEK293 cell (green and red channel with or without detrending) 5.4 min after injection. Pseudocolor indicates photon counts at each pixel. Correlation curves are obtained by calculating correlation in the ROI indicated as squares in the images.

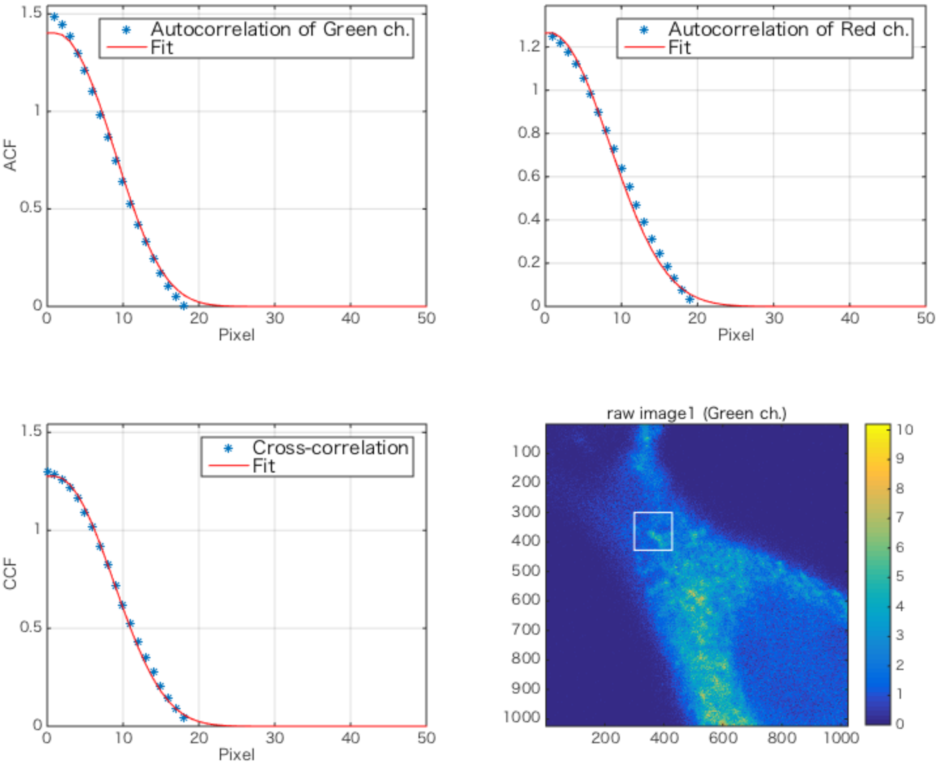

Supplement Figure S4.

Correlation functions (symbol) and its fitting results (red line) of HEK293 cell (5.4 min) in the ROI at the position of squares in the image.

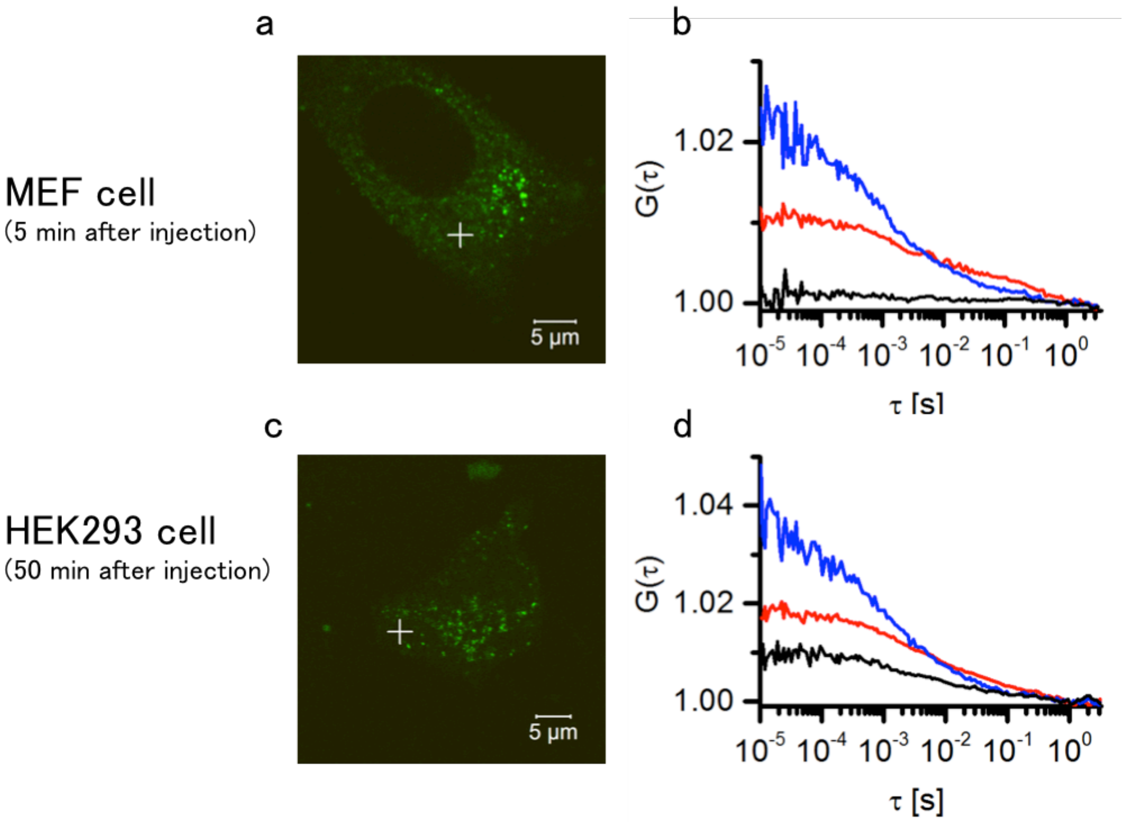

Supplement Figure S5.

Fluorescence cross-correlation spectroscopy (FCCS) analysis of DNA degradation in MEF and HEK293 cell cytoplasm. (a) Fluorescence image of MEF cell 5 min after DNA probe injection. (b) Autocorrelation curves of green channel (blue), red channel (red) and cross-correlation curve (black) measured at the point of cross in (a). (c) Fluorescence image of HEK293 cell 50 min after DNA probe injection. (d) Autocorrelation curves of green channel (blue), red channel (red) and cross-correlation curve (black) measured at the point of cross in (c).
